# *Lawsonella clevelandensis* breast abscess in a young woman with uncontrolled type 1 diabetes: a case report

**DOI:** 10.3389/fgwh.2026.1945144

**Published:** 2026-09-09

**Authors:** Weihong Chen, Nanhong Su, Qingjiang Lin, Longyan Ke, Shumin Xie, Jiakang Bai, Xuchun Bai

**Affiliations:** 1 Department of Clinical Medical Research Center, Anxi County Hospital, Quanzhou, Fujian, China; 2 Department of Clinical Laboratory, Anxi County Hospital, Quanzhou, Fujian, China; 3 Department of Graduate School, Capital Medical University, Beijing, China; 4 Department of Emergency, Anxi County Hospital, Quanzhou, Fujian, China

**Keywords:** 16S rRNA sequencing, breast abscess, case report, *lawsonella clevelandensis*, type 1 diabetes mellitus

## Abstract

**Background:**

*Lawsonella clevelandensis* (*L. clevelandensis*) is a recently described anaerobic, partially acid-fast Gram-positive bacillus that has been increasingly recognized as an opportunistic pathogen causing abscess formation in immunocompromised hosts. However, breast abscess caused by this organism in young diabetic patients is rarely reported.

**Case presentation:**

A 22-year-old woman with a 5-year history of poorly controlled type 1 diabetes mellitus presented with a one-week history of left breast pain and erythema. Ultrasound revealed mastitis with abscess formation. Needle aspiration yielded purulent material that was partially acid-fast on staining but negative for Mycobacterium tuberculosis complex PCR. Conventional aerobic culture showed no growth, while anaerobic culture yielded colonies after 7 days of incubation. The isolate was identified as *L. clevelandensis* by 16S rRNA gene sequencing (99.722% similarity). The patient received percutaneous drainage, antibiotics, and intensive insulin therapy, with marked clinical improvement.

**Conclusion:**

This case highlights the diagnostic challenges posed by *L. clevelandensis* due to its fastidious growth and misleading acid-fast positivity. It should be suspected in diabetic patients with breast abscesses when conventional cultures are negative and tuberculosis is excluded. Prolonged anaerobic culture and molecular methods are essential for definitive diagnosis.

## Background

*Lawsonella clevelandensis* is a fastidious, anaerobic, partially acid-fast Gram-positive bacillus belonging to the suborder Corynebacterineae within the phylum Actinobacteria (1). First described by Melissa et al (2). in 2013 and formally established as a novel genus and species by Bell et al (3). in 2016, this organism was isolated from human abscesses at various anatomical sites. The bacterium exhibits distinctive microbiological features, including slow growth under anaerobic or microaerophilic conditions, Gram-variable staining with a tendency to decolorize, and partial acid-fast positivity due to the presence of short-chain mycolic acids in its cell wall (4). These characteristics often lead to diagnostic confusion with other acid-fast organisms such as Mycobacterium species, Nocardia, and non-tuberculous mycobacteria, particularly in routine clinical laboratory practice (5).

Clinically, *L. clevelandensis* has been increasingly recognized as an opportunistic pathogen capable of causing monomicrobial abscess formation in various body sites, including the breast, liver, abdomen, spine, and vascular grafts (6). Most reported cases occur in patients with diabetes, obesity, malignancy, or recent surgery. The organism is a commensal of human skin and mucosal surfaces, and disruption of cutaneous barriers or local immunosuppression may facilitate bacterial translocation and abscess formation.

*L. clevelandensis* is difficult to identify by conventional methods due to its slow anaerobic growth, Gram-variable staining, and misleading partial acid-fast positivity, which often directs diagnostic workup toward tuberculosis or nocardiosis (7). To date, only a limited number of *L. clevelandensis* infections have been documented worldwide, and breast abscess cases remain exceedingly rare (8). Here, we report a case of breast abscess caused by *L. clevelandensis* in a young woman with poorly controlled type 1 diabetes mellitus, highlighting the diagnostic pitfalls and therapeutic considerations associated with this emerging pathogen.

## Case report

### Patient presentation and initial evaluation

A 22-year-old woman presented to the Emergency Department of Anxi County Hospital on March 7, 2026, with a one-week history of progressive pain in the left breast. The pain was persistent, localized to the nipple and periarcolar region, without radiation to other areas. She denied fever, cough, sputum production, chest tightness, nausea, or vomiting. Her past medical history was significant for type 1 diabetes mellitus diagnosed five years earlier, for which she had been receiving insulin therapy with irregular adherence. She had no history of breast trauma, surgery, or lactation. This study was approved by the Ethics Committee of Anxi County Hospital (Approval Number: 20260041). Written informed consent was obtained from the patient for the publication of this case report and any accompanying images.

On admission, vital signs were as follows: temperature 36.2 °C, pulse 89 beats/min, respiratory rate 20 breaths/min, and blood pressure 138/89 mmHg. Physical examination revealed an asymmetrical chest with obvious erythema and swelling over the left breast. A firm, tender mass measuring approximately 5.0 × 2.5 × 5.0 cm was palpable in the lower aspect of the left breast, with ill-defined borders, reduced mobility, and no ulceration or peau d'orange change. The nipple showed no obvious retraction. A slightly enlarged lymph node, approximately 1.5 × 1.0 cm in size, was palpable in the left axilla, which was soft, well-defined, and non-tender. No other superficial lymphadenopathy was detected.

Initial laboratory investigations revealed poor glycemic control with a random blood glucose of 19.80 mmol/L and glycated hemoglobin (HbA1c) of 14.10%. Inflammatory markers were modestly elevated: white blood cell count 10.16 × 10⁹/L, C-reactive protein 7.36 mg/L, procalcitonin 0.07 ng/mL, and interleukin-6 5.2 pg/mL. Biochemical analysis showed hypoalbuminemia (albumin 26.5 g/L) and hypoproteinemia (total protein 61.7 g/L). Other parameters, including coagulation profile, were within normal limits except for an elevated fibrinogen level of 5.22 g/L.

### Radiological findings

**Figure 1:**
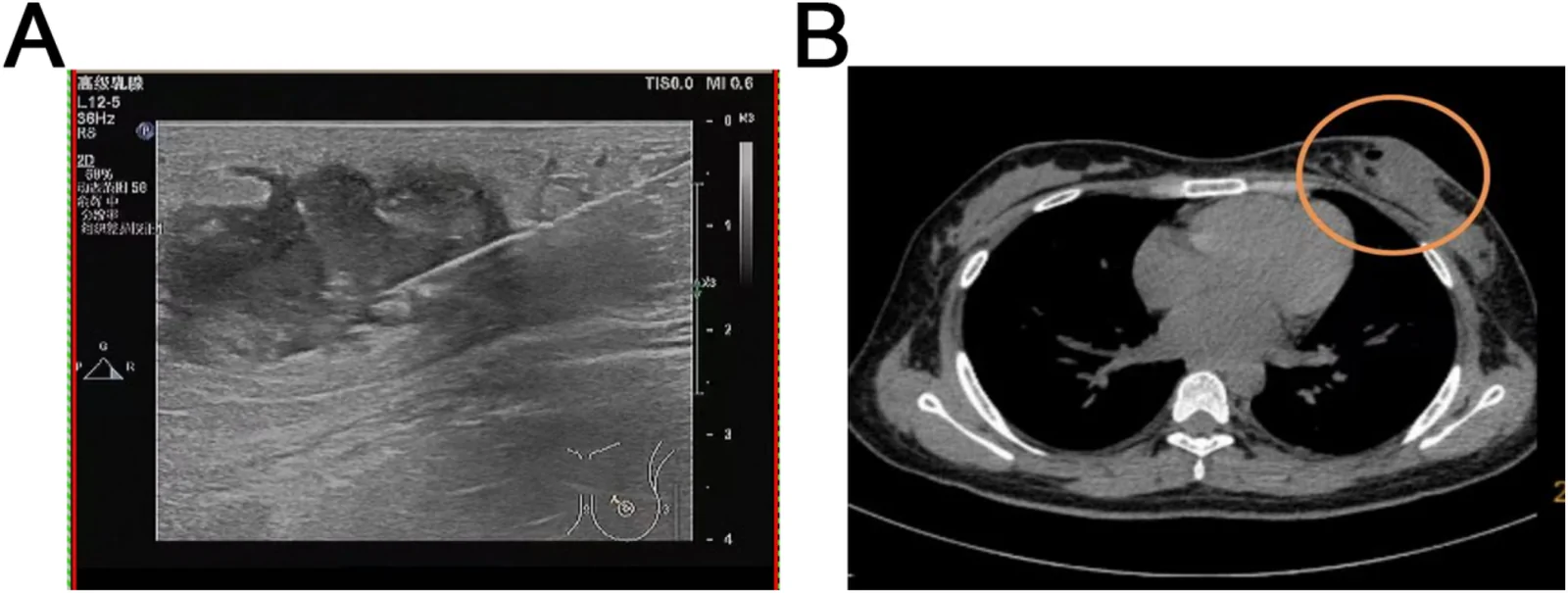
Radiological findings of the left breast lesion. **(A)** Breast ultrasound demonstrating a mixed echogenic lesion measuring approximately 5.1 × 5.0 × 2.3 cm with ill-defined borders, irregular shape, and heterogeneous internal echoes, suggestive of mastitis with abscess formation (BI-RADS category 3). Left axillary lymphadenopathy was also noted. **(B)** Chest computed tomography (CT) showing enlargement of the left breast with multiple scattered punctate gas densities (arrows) in the medial aspect, with surrounding fat stranding and left axillary lymphadenopathy, consistent with suppurative mastitis.

Ultrasound examination of the breasts on March 7, 2026, demonstrated a mixed echogenic lesion in the left breast, measuring approximately 5.1 × 5.0 × 2.3 cm, with ill-defined borders, irregular shape, heterogeneous internal echoes, and mobile punctate echoes upon compression, suggestive of mastitis with abscess formation (BI-RADS category 3). No significant abnormality was detected in the right breast. Left axillary lymphadenopathy was also noted (Figure 1A). Chest computed tomography (CT) performed on the same day revealed enlargement of the left breast with multiple scattered punctate gas densities in the medial aspect, with surrounding fat stranding and left axillary lymphadenopathy, consistent with suppurative mastitis (Figure 1B). The clinical course is summarized in Table 1.

**Table 1:** Clinical timeline of the diagnostic and treatment course.

Date	Clinical event
Day 1 (Mar 7, 2026)	Symptom onset: left breast pain and erythema; presented to Emergency Department; ultrasound and CT performed.
Day 3 (Mar 9, 2026)	Needle aspiration performed; acid-fast staining positive (4+); TB-PCR negative.
Day 10 (Mar 16, 2026)	Anaerobic culture yielded colonies after 7 days of incubation.
Day 12 (Mar 18, 2026)	16S rRNA sequencing identified *L. clevelandensis*; targeted antibiotic therapy initiated.
Day 19 (Mar 25, 2026)	Significant clinical improvement; patient transferred for definitive surgical management.

### Microbiological and molecular diagnosis

**Figure 2:**
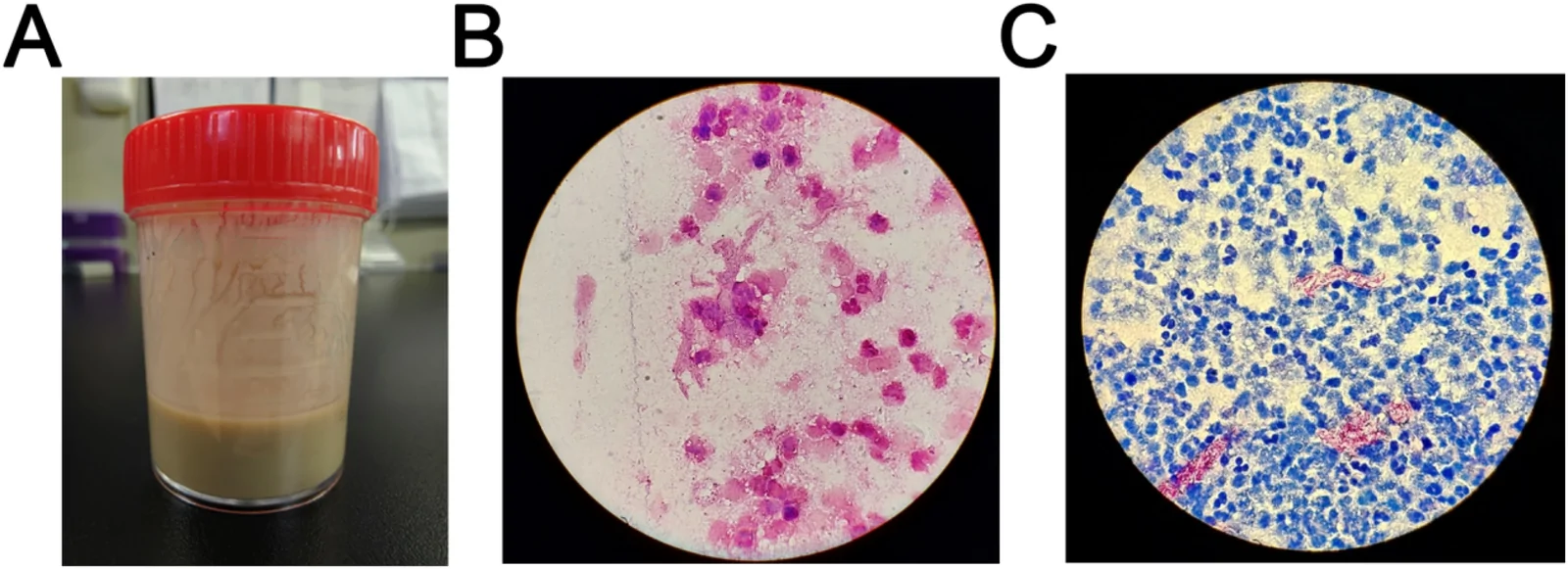
Gross appearance and microscopic features of the aspirated material. **(A)** Greenish-purulent fluid aspirated from the left breast abscess via ultrasound-guided percutaneous needle aspiration. **(B)** Gram staining of the aspirate revealing Gram-positive bacilli with pale purple coloration (original magnification  × 1000). **(C)** Acid-fast staining of the aspirate demonstrating positive (4+) acid-fast bacilli (red) against a blue background (original magnification   ×  1000).

On March 9, 2026, ultrasound-guided percutaneous needle aspiration and biopsy of the left breast lesion were performed. Approximately 15 mL of greenish-purulent fluid was aspirated, and tissue cores were obtained for histopathological examination (Figure 2A). Gram staining of the aspirate revealed Gram-positive bacilli with pale purple coloration (Figure 2B), while acid-fast staining was positive (4+), showing more than 10 acid-fast bacilli per oil immersion field (Figure 2C).

**Figure 3:**
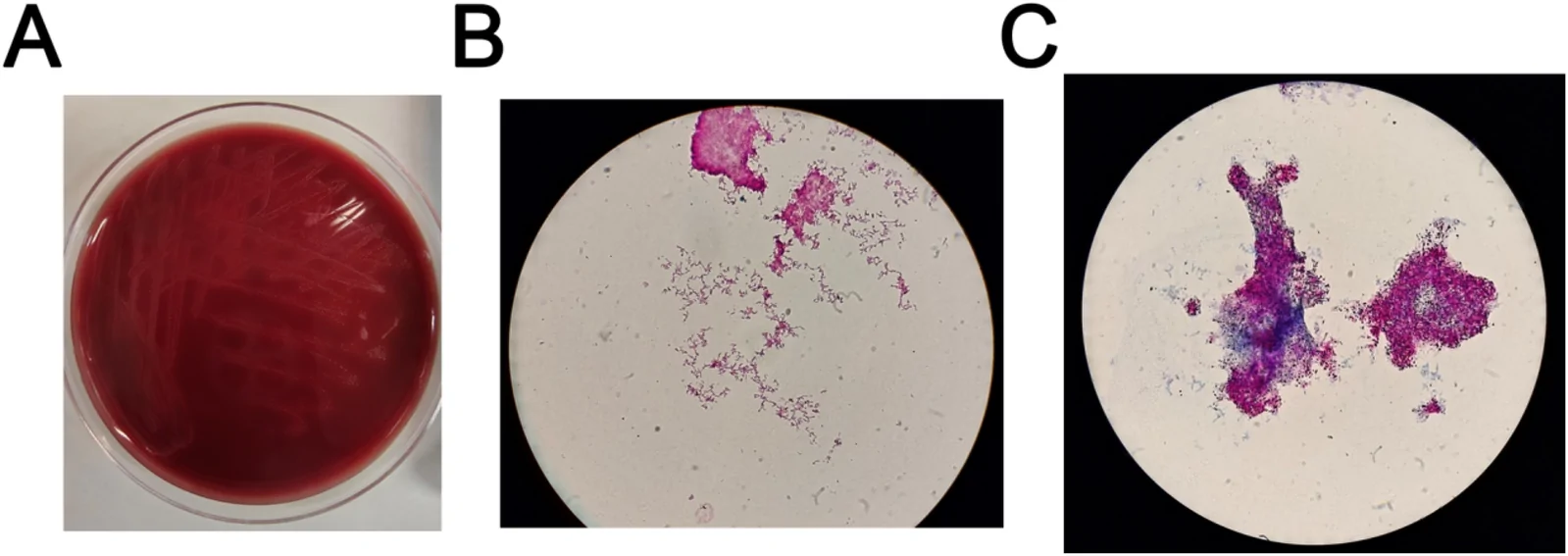
Anaerobic culture and staining characteristics of the isolated organism. **(A)** Tiny, translucent colonies on Columbia blood agar after 7 days of anaerobic incubation at 37 °C. **(B)** Gram staining of the colonies revealing Gram-positive bacilli with a tendency to decolorize, appearing as rod-shaped organisms arranged singly, in short chains, or in clusters (original magnification   ×  1000). **(C)** Acid-fast staining of the colonies confirming acid-fast positivity (red bacilli) (original magnification   ×  1000).

However, nucleic acid amplification testing for the Mycobacterium tuberculosis complex returned negative. Aerobic bacterial culture of the aspirate showed no growth after 5 days of incubation. Concurrently, anaerobic culture on Columbia blood agar incubated at 37 °C yielded tiny, translucent colonies after 7 days (Figure 3A). Subculture and staining of the colonies revealed Gram-positive bacilli, which were prone to decolorization resulting in Gram-variable appearance, and were observed as rod-shaped organisms arranged singly, in short chains, or in clusters (Figure 3B). The organisms remained acid-fast positive (Figure 3C).

**Figure 4:**
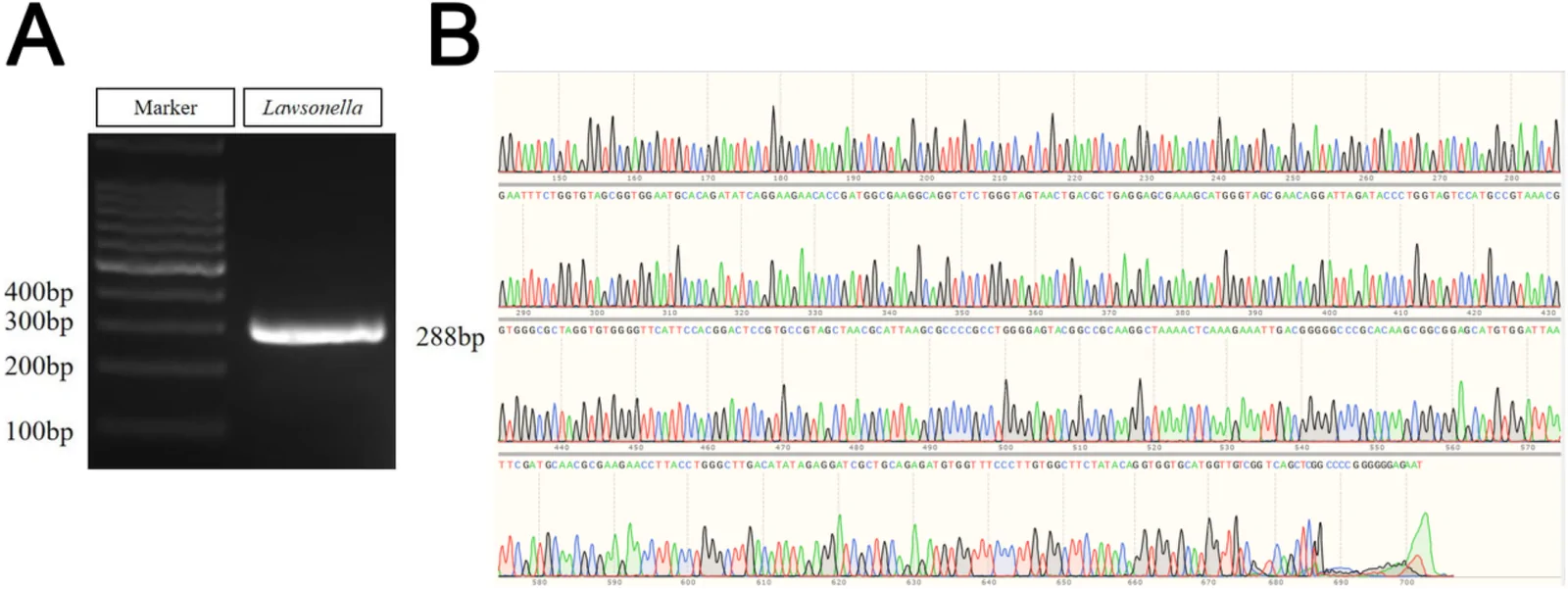
Molecular identification of the isolate. **(A)** Agarose gel electrophoresis of PCR products amplified using species-specific primers Lawsonella-F and Lawsonella-R, showing a specific band of approximately 288 bp. Lane M: DNA molecular weight marker; lane 1: clinical isolate. **(B)** BLAST alignment results demonstrating 99.722% identity to the reference strain *Lawsonella clevelandensis* (NR151867.1) and 95.978% identity to *Dietzia alimentaria* (NR117306.1), confirming the identification of the isolate as *L. clevelandensis*.

For definitive identification, bacterial genomic DNA was extracted from the isolate, and PCR amplification of the 16S rRNA gene was performed using the species-specific primer pair Lawsonella-F (5'-CGAGCGTTGTCCGGAATTAC-3’) and Lawsonella-R (5'-CACCTAGCGCCCACCGTTTA-3’), yielding a 288-bp amplicon (Figure 4A). The PCR products were sequenced and subjected to BLAST analysis against the NCBI GenBank database. The sequence showed 99.722% similarity to the reference strain *Lawsonella clevelandensis* (NR151867.1), while the next closest match was *Dietzia alimentaria* (NR117306.1) with only 95.978% similarity, confirming the identity of the isolate as *L. clevelandensis* (Figure 4B and Table 2).

**Table 2:** BLAST analysis of the 16S rRNA gene sequence.

Species	Accession	Similarity
*Lawsonella clevelandensis*	NR151867.1	99.722%
*Dietzia alimentaria*	NR117306.1	95.978%

### Treatment and clinical course

Upon admission, the patient was initiated on empirical antibiotic therapy with levofloxacin 100 mL (0.5 g) intravenously once daily, in combination with dexamethasone 5 mg intravenously once daily for its anti-inflammatory effect. However, given the lack of clinical response and the subsequent identification of *L. clevelandensis* as the causative pathogen, the antibiotic regimen was adjusted accordingly to amoxicillin-clavulanate 1.2 g intravenously every 8 h for 14 days. Intensive insulin therapy was instituted, consisting of insulin aspart 10 units before each meal and insulin detemir 15 units at bedtime for glycemic control.

The patient's left breast pain improved substantially following percutaneous drainage, antibiotic therapy, and glycemic management. By the time of transfer, physical examination showed marked reduction in breast erythema and swelling, with the palpable mass decreasing to approximately 3.0 × 2.5 × 3.0 cm. The left axillary lymph node remained palpable but was reduced in size and non-tender. The patient was subsequently transferred to a higher-level hospital for definitive surgical management. At the time of transfer, her local inflammatory signs had significantly subsided, and systemic symptoms had resolved.

## Discussion

The strong acid-fast positivity (4+) observed in this case, coupled with a negative TB-PCR, exemplifies the diagnostic confusion these organisms cause. *L. clevelandensis* is microbiologically characterized as partially acid-fast due to the presence of short-chain mycolic acids in its cell wall, although the staining intensity in clinical specimens can appear strong (4+). This diagnostic dilemma is a recurring theme in *L. clevelandensis* infections, as the organism's partial acid-fast positivity frequently misdirects diagnostic workup toward tuberculosis or nocardiosis. This misleading staining property, combined with the organism's strict anaerobic requirements and slow growth, contributes to diagnostic delays (9). In a recent case report describing a 46-year-old woman with a 6-year history of recurrent breast abscess, the patient was initially treated with quadruple anti-tuberculosis therapy for nearly 5 years before metagenomic sequencing finally identified *L. clevelandensis* as the causative pathogen. This case powerfully illustrates the clinical consequences of misdiagnosis and underscores the importance of molecular diagnostic methods in achieving accurate identification (10).

The microbiological features of *L. clevelandensis* present substantial obstacles to conventional laboratory identification. The organism requires prolonged anaerobic incubation, typically 5 to 7 days, to produce visible colonies (11). This growth pattern mirrors that reported in other cases, where colonies typically appear on enriched solid media such as Brucella blood agar or phenylethyl alcohol agar within 4 to 14 days. Notably, a recent case demonstrated that *L. clevelandensis* can also be maintained in thioglycolate broth, with growth observed within 21 to 28 days—a finding that extends our understanding of the organism's cultivation requirements (12). Routine identification methods, including MALDI-TOF mass spectrometry, have consistently failed to identify *L. clevelandensis* due to the absence of reference spectra in commercial databases, making 16S rRNA gene sequencing or metagenomic next-generation sequencing (mNGS) essential for definitive diagnosis (13).

The clinical presentation of *L. clevelandensis* breast abscess appears to occur predominantly in patients with underlying comorbidities. Among the reported cases of breast abscess caused by this organism, including the present case, the majority occurred in patients with diabetes mellitus and/or obesity. Cases described in the literature include patients with type 2 diabetes mellitus, end-stage renal disease, and obesity, while others had no identifiable risk factors. In one case, infection developed following autologous fat grafting for breast augmentation, suggesting that local tissue manipulation may serve as a predisposing factor (14). To our knowledge, this 22-year-old patient with poorly controlled type 1 diabetes mellitus (HbA1c 14.10%) is the youngest reported case of *L. clevelandensis* breast abscess to date. Rather than age, the primary predisposing factor was likely her immunocompromised state due to chronic hyperglycemia—reinforcing the role of impaired glycemic control in creating a permissive niche for this opportunistic pathogen. The extremely elevated HbA1c in our case suggests that poor glycemic control may be a particularly strong risk factor for *L. clevelandensis* infection. In this patient, chronic hyperglycemia was the likely driver of such translocation. consistent with the organism's proposed role as a commensal of skin and mucosal surfaces that translocates when local or systemic host defenses are compromised.

*L. clevelandensis* is highly susceptible to a broad range of antibiotics, including *β*-lactams, macrolides, tetracyclines, fluoroquinolones, glycopeptides, and oxazolidinones, consistent with the absence of known resistance genes by whole-genome sequencing. Amoxicillin-clavulanate has been the most frequently employed oral agent in reported breast abscess cases, with generally favorable outcomes (15). In our case, the patient was initially treated with levofloxacin empirically. Following identification of *L. clevelandensis*, antibiotic therapy was adjusted accordingly. The clinical improvement observed supports the organism's susceptibility to appropriate antimicrobial agents.

Source control remains a cornerstone of management for *L. clevelandensis* abscesses. In nearly all reported cases, surgical or percutaneous drainage was performed, often in conjunction with antibiotic therapy. In our patient, ultrasound-guided percutaneous needle aspiration provided effective initial drainage, resulting in substantial symptomatic improvement (16). This approach aligns with the current literature, which emphasizes that timely debridement and drainage, combined with appropriate antibiotic therapy, can usually lead to cure. The recurrence rate appears low when adequate source control is achieved, although prolonged antibiotic courses may be necessary in selected cases.

Several limitations of this case report should be acknowledged. First, the patient was transferred to a higher-level hospital for definitive surgical management, precluding long-term follow-up at our institution. Second, antimicrobial susceptibility testing of the isolated strain was not performed, limiting our ability to provide genotype-phenotype correlation data. Third, the absence of a comparative assessment of different molecular diagnostic platforms (e.g., 16S rRNA sequencing versus mNGS) in our case represents a missed opportunity to evaluate their relative performance in this specific clinical context.

## Conclusion

This case demonstrates that *L. clevelandensis* should be considered in diabetic patients with breast abscesses, particularly those with poor glycemic control, when acid-fast staining is positive but tuberculosis testing is negative. Prolonged anaerobic culture and molecular methods such as 16S rRNA sequencing or mNGS are essential for definitive diagnosis. Increased awareness of this emerging pathogen is crucial to prevent diagnostic delays and improve patient outcomes.

## Data Availability

The original contributions presented in the study are included in the article/Supplementary Material, further inquiries can be directed to the corresponding author.
